# TACI and BTK gene analysis in predominantly antibody deficiencies among the primary immunodeficiency disorder patients in Bangladesh

**DOI:** 10.3389/fmed.2025.1569810

**Published:** 2025-09-25

**Authors:** Tripty Chakrobortty, Chandan Kumar Roy, Mohammad Imnul Islam, Ismet Niger, Kamrul Laila, Avizit Sarker, Nusrat Akhtar Juyee, Md. Mydul Islam, Fahmida Hoque, Partho Protim Chakrobortty, S. M. Ali Ahmed, Abu Naser Ibne Sattar

**Affiliations:** ^1^Department of Microbiology, Jashore Medical College, Jashore, Bangladesh; ^2^Department of Microbiology and Immunology, Bangabandhu Sheikh Mujib Medical University, Shahbag, Dhaka, Bangladesh; ^3^Department of Paediatrics, Bangabandhu Sheikh Mujib Medical University, Shahbag, Dhaka, Bangladesh; ^4^Department of Microbiology, Dhaka Medical College, Dhaka, Bangladesh; ^5^Department of Microbiology, Comilla Medical College, Cumilla, Bangladesh; ^6^Department of Microbiology, Jamalpur Medical College, Jamalpur, Bangladesh; ^7^Department of Surgery, Magura 250-bed General Hospital, Magura, Bangladesh

**Keywords:** PIDDs, PAD, Btk, TACI/TNFRSF13B, sanger sequencing

## Abstract

**Background:**

Common variable immune deficiency disorder (CVID) and X-linked agammaglobulinemia (XLA) are the most prevalent predominantly antibody deficiencies (PADs). Analysis of the TACI/TNFRSF13B gene in CVID and BTK genes in XLA patients using Sanger sequencing can help make specific diagnoses of these cases. The study aimed to find the TACI and BTK gene mutations and their allelic variations associated with CVID and XLA patients.

**Methods:**

This cross-sectional study was conducted on clinically suspected PAD patients who attended the Department of Pediatrics, Bangabandhu Sheikh Mujib Medical University (BSMMU), Bangladesh, from September 2022 to August 2023. Serum immunoglobulin levels, immunophenotyping by flow cytometry, and PCR were conducted in the Department of Microbiology and Immunology at BSMMU. Genetic analysis of the TACI and BTK genes was conducted using Sanger sequencing at DNA Solutions Limited, Dhaka, Bangladesh. The sequencing results were validated using the NCBI GenBank.

**Results:**

Of 35 clinically suspected PAD patients, 15 (42.86%) were diagnosed as PAD patients. Within this group, seven (46.67%) were diagnosed with CVID, seven (46.67%) with XLA, and one (6.66%) with agammaglobulinemia other than XLA. The analysis of the TACI gene revealed no pathogenic variants in the CVID patients. Upon analyzing exons 2 to 19 of the BTK gene, seven pathogenic/likely pathogenic mutations were detected, consisting of four nonsense and three missense mutations. Among these, three were found to be novel mutations, including two missense and one nonsense mutation.

**Conclusion:**

The genetic analysis of the TACI gene in CVID patients revealed no pathogenic variants. The BTK gene displayed heterogeneous mutations, with nonsense mutations being the most prevalent. In this cohort, XLA patients presented three *de novo* point mutations.

## Introduction

Primary immune deficiency disorders (PIDDs) are a diverse set of hereditary diseases that impair various aspects of the innate and adaptive immune systems. Around the world, primary immunodeficiencies, also known as inborn errors of immunity (IEI), are estimated to affect approximately 6 million people, with 70 to 90% remaining undiagnosed and untreated (1, 2). The International Union of Immunological Societies (IUIS) is an international group of experts that evaluates these diseases every 2 years based on their clinical and immunologic phenotypes. The 2022 IUIS classification revealed 485 illnesses, including 55 novel monogenic defects and one autoimmune phenocopy. In the 2024 update, this number increased to 559 inborn errors of immunity (IEI), including 67 novel monogenic defects and two phenocopies (3, 4). According to the most fundamental categories for immunologic illnesses, the most prevalent PIDDs are predominantly antibody deficiencies (PAD), which has multiple etiologies (5).

There is relatively little information available on PIDDs in Bangladesh. In 2016, the results of a study conducted at a tertiary care hospital in Bangladesh showed that the majority (60%) of patients had PADs, followed by combined immune deficiencies (30%) and phagocytic disorders (10%). Among the most prevalent PADs, transient hypogammaglobulinemia of infancy accounted for 33.33%, followed by 16.66% each for common variable immune deficiency disorder (CVID), agammaglobulinemia, selective IgM deficiency (SIgMD), and selective IgA deficiency (SIgAD) (6). Another study (7) also showed that the majority (64.28%) of PIDD cases were PAD patients, with CVID and agammaglobulinemia being the most common antibody deficiency disorders (21.43%).

The etiology of the vast majority of CVID cases is unknown. Since 2003, mutations in four genes—ICOS, CD19, BAFF-R, and TNFRSF13B (encoding TACI, the transmembrane activator, calcium modulator, and cyclophilin ligand interactor)—have been identified as being responsible for 10–15% of CVID cases (8–14). With one or two mutant alleles, TACI deficiency has been identified in up to 10% of CVID patients (10, 11). For the specific diagnosis of CVID, mutations in TACI/TNFRSF13B can be detected by nucleotide sequencing (15).

In cases of X-linked agammaglobulinemia (XLA), is prevalent immunodeficiency disorder in Bangladesh, patients have between 0.01 and 0.5% CD19^+^ B lymphocytes in their blood. These cells show high levels of surface IgM expression (16). The absence or reduction of intracellular BTK protein detection by flow cytometry can aid in diagnosing XLA (17). Approximately 95% of XLA patients have markedly reduced or absent BTK protein expression, but 5% may have normal protein expression with abnormal function (18). These 5% of patients can also be diagnosed through mutation analysis of the BTK gene. Therefore, BTK gene mutation analysis using nucleotide sequencing can be used for the specific diagnosis of this disorder (19).

Autosomal recessive agammaglobulinemia is an extremely rare condition, accounting for only 10 to 15% of agammaglobulinemia patients. It is a genetically diverse illness characterized by a significant decrease in all antibody classes and an absence of peripheral B cells, with no BTK mutations (20, 21).

The 2024 update of the IUIS guides the creation of panels used for targeted gene sequencing to aid in the clinical genetic diagnosis of IEI and is meant to serve as a follow-up resource for researchers and clinicians (22). Different countries are using nucleotide sequencing for the diagnosis of PIDDs worldwide, as genetic testing has assumed increasing importance in the diagnosis and management of PIDDs (23). To date, nucleotide sequencing has not been used for the diagnosis of PIDDs in Bangladesh. The aim of this study was to assess variants in the TACI/TNFRSF13B and BTK genes using Sanger sequencing to enable the specific diagnosis of CVID and XLA cases among PAD patients in Bangladesh.

## Materials and methods

Among 35 clinically suspected PAD patients, 15 were diagnosed as PAD patients and enrolled in this cross-sectional study conducted from September 2022 to August 2023. Among them, nine clinically diagnosed PAD patients were selected from the registry of the Pediatrics Department at Bangabandhu Sheikh Mujib Medical University (BSMMU). A total of six were diagnosed as PAD patients among 26 newly suspected PIDD patients who attended the Department of Pediatrics at Ad-din Hospital, Dhaka. Patients were enrolled in this study based on the standard criteria of the Jeffrey Modell Foundation (24), along with the complete blood count, serum immunoglobulin (IgM, IgG, IgA, IgE) levels, and flow cytometric analysis of peripheral blood for T-B-NK cell surface markers. The age group of the patient was defined as between 0 and 18 years old, according to the United Nations Convention on the Rights of the Child. Informed written consent was obtained from the parents, and prior approval was obtained from the Institutional Review Board of BSMMU, Dhaka, Bangladesh. (Ref. no. BSMMU/2022/12963; Date: 31.12.2022) was obtained before enrollment in this study.

Flow cytometry-based immunophenotyping was performed using a BECKMAN COULTER DxFLEX Flow Cytometer (Cat. No. 651155) with monoclonal antibodies (Abcam, UK; Beckman Coulter, USA; BioLegend, USA; and BD Biosciences, USA). The BD FACSuite™ software was used for data acquisition and analysis.

BTK protein expression was assessed in the monocytes of agammaglobulinemia patients who showed reduced levels (<2 SD) of serum immunoglobulin classes compared to the age-specific normal level (25), and B cells were absent or markedly reduced (<2% of circulating B cells). Age-specific reference values for the above-mentioned proteins have not yet been established by the ESID guidelines (November, 2019). A healthy control of the same age group was included for each case to compare the mean fluorescence intensity (MFI) of BTK proteins of diagnosed XLA cases with those of healthy donors (18). Samples from both suspected PAD patients and healthy controls were analyzed simultaneously in two different test tubes for each protein, and the events were compared between the suspected case and the healthy donor using a histogram. The mean fluorescence intensity (MFI) of the anti-BTK antibody was measured and compared with the MFI of the healthy control sample (Supplementary file 1).

Serum IgG, IgM, and IgA levels were determined using an automated nephelometry analyzer (SIEMENS Atellica NEPH 630; Cat. No. 191227), and IgE was assessed using a chemiluminescence auto-analyzer (SIEMENS Advia Centaur XPT; Cat. No. 1392409), as per the manufacturer’s instructions.

The TACI/TNFRSF13B and BTK genes were amplified using conventional PCR from a peripheral venous blood specimen. PCR assays were performed on a Proflex PCR system (Applied Biosystems, Thermo Fisher Scientific, USA) in the PCR laboratory of the Department of Microbiology and Immunology, BSMMU. Genomic DNA extraction from peripheral venous blood was performed according to the manufacturer’s instructions (TRUPCR® BLOOD DNA EXTRACTION KIT, Kilpest India Ltd., India). As the Department of Microbiology and Immunology at BSMMU lacks the setup for DNA sequencing, this procedure was carried out at DNA Solution Limited, Shyamoli, Dhaka. The nucleotide sequence was determined from the final PCR products using the Sanger dideoxy method with PCR primers on a 3,500 DX Genetic Analyzer (Thermo Fisher Scientific, USA).

The collected data were checked, edited, and analyzed using SPSS software package version 27 (Strata Corporation, College Station, Texas). All diagnosed cases were categorized into three groups: CVID, XLA, and agammaglobulinemia other than XLA, according to serological tests (IgG, IgM, IgA, and IgE), basic T-B-NK cell markers, CD27 and IgD markers, and BTK protein expression. After obtaining the results of Sanger sequencing, the obtained data were analyzed using various editing software tools. Sequence chromatograms from 14 patients were edited and converted to FASTA format in Chromas software. Reference sequences of the exons of the TACI and BTK genes were obtained from the NCBI GenBank. Mutation analysis was performed through multiple sequence alignment using MEGA11, applying the ClustalW Multiple Alignment algorithm. Nucleotide and amino acid positions were numbered according to the cDNA sequence (26). For the annotation of the mutation, the functional implication of the missense variants was assessed using the computational algorithm PolyPhen2.

## Results

This cross-sectional study was conducted on 35 clinically suspected PAD patients; among them, 15 (42.86%) were diagnosed with PAD based on laboratory test results.

**Figure 1:**
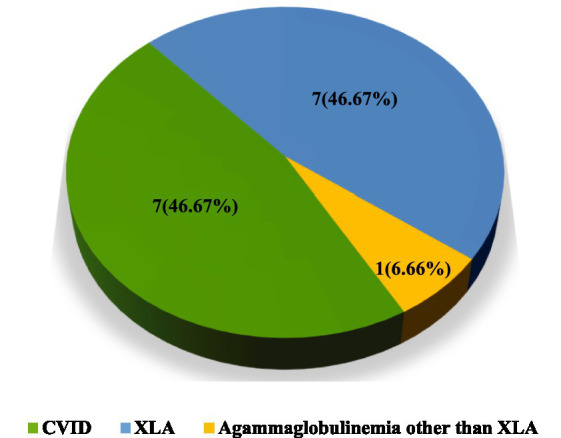
Distribution of the types of PAD patients (*n* = 15).

Of the 15 laboratory-confirmed PAD patients, seven (46.67%) were diagnosed with CVID and eight (53.33%) with agammaglobulinemia. Among the agammaglobulinemia patients, seven (46.67%) had XLA, while one (6.66%) was diagnosed as a case of agammaglobulinemia other than XLA (Figure 1).

Table 1 shows the important demographic, clinical, and laboratory findings of the diagnosed CVID patients. Switched memory B cells (CD19 + CD27 + IgD-) were decreased below the age-related reference value (27, 28). All patients had decreased serum IgG levels, along with reduced levels of either serum IgA or IgM, except for two patients (P4 and P5) who were receiving IVIG at the time of evaluation. In P3, the serum IgG level, along with switched memory B cells, was found to be decreased.

**Table 1:** Demographic, clinical, and laboratory findings of the diagnosed CVID patients.

Pt’s no.	Sex	Age	Age of onset	History of infection	CD19^+^ B cell (%)	CD19^+^ CD27^+^IgD^−^ (%)	Serum IgM (gm/l)	Serum IgG (gm/l)	Serum IgA (gm/l)
P1	F	11y	1y	Fever, RTI	20.0 (17–37.2)	**2.0** (2.9–17.4)	2.50 (0.41–2.55)	**<1.34** (5.03–17.19)	**<0.26** (0.42–2.95)
P2	F	7y	2y	Fever, RTI	**15.6** (17–37.2)	**1.8** (2.9–17.4)	**0.30** (0.38–2.51)	**1.38** (4.62–16.82)	0.80 (0.34–2.74)
P3	M	1y	3 m	Pneumonia	58.0 (12.9–29.2)	**0.51** (0.6–3.7)	1.57 (0.40–1.32)	**<1.34** (1.64–5.88)	0.36 (0.16–0.50)
P4	M	6y	1y	Fever, RTI	25.0 (17–37.2)	**2.0** (2.9–17.4)	**0.32** (0.37–2.24)	10.5 (3.86–14.70)	1.06 (0.29–2.56)
P5	M	12y	1y	Fever, pneumonia	**4.0** (17–37.2)	**2.7** (2.9–17.4)	**0.38** (0.41–2.55)	14.0 (5.03–17.19)	**0.26** (0.42–2.95)
P6	M	1y	7 m	Fever, RTI	**4.7** (12.9–29.2)	**0.56** (0.6–3.7)	1.02 (0.40–1.43)	**2.00** (2.46–9.04)	**0.26** (0.27–0.66)
P7	M	13y	6y	Fever, RTI	16.7 (11.9–21)	**0.64** (2.9–17.4)	**0.22** (0.41–2.55)	**1.62** (5.03–17.19)	**0.26** (0.42–2.95)

y: year; m: month; F: female; M: male. The bold values indicate significant results.

**Figure 2:**
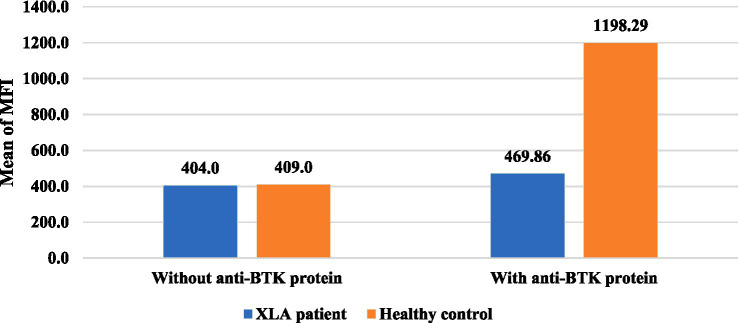
Distribution of intracellular BTK protein expression among the XLA patients. The mean fluorescence intensity (MFI) of BTK protein expression in the healthy controls (*n* = 7) was 1198.29 and in XLA patients (*n* = 7) was 469.86, which was markedly reduced compared to that of the healthy control after stimulation with an anti-BTK monoclonal antibody. *AG: agammaglobulinemia other than XLA.

**Figure 3:**
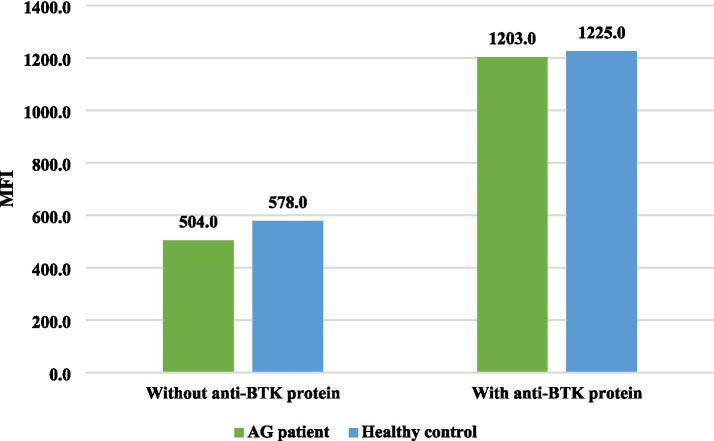
Distribution of intracellular BTK protein expression in agammaglobulinemia patient, other than the XLA patient. The MFI of BTK protein expression in the healthy control (*n* = 1) was 1,225 and in the agammaglobulinemia other than XLA patient (*n* = 1) was 1,203, which was very close to that of the healthy control after stimulation with an anti-BTK monoclonal antibody. *AG: agammaglobulinemia other than XLA.

Table 2 shows some pivotal demographic, clinical, and laboratory findings of the diagnosed agammaglobulinemia patients. The CD19 + B cell count was decreased (<2SD) in all seven XLA patients. Serum IgG, IgA, and IgM levels were also decreased in all of these patients below the age-related reference value. BTK protein expression was markedly reduced after stimulation with an anti-BTK monoclonal antibody in all these patients. In P9 and P12, there was a positive family history of maternal male relatives who died in early childhood due to respiratory tract infections. The rest of the patients had no such history. As P14 had no maternal uncle, information regarding the death of maternal male relatives could not be obtained. On the other hand, one patient with agammaglobulinemia other than XLA showed decreased CD19 + B cells, serum IgA, and IgM levels, but BTK protein expression was very close to that of the healthy control after stimulation with an anti-BTK monoclonal antibody. Serum IgG was normal, as the patient was receiving IVIG at the time of evaluation. The comparison of intracellular BTK protein expression among the XLA patients and agammaglobulinemia patients with healthy donors is shown in Figures 2, 3.

**Table 2:** Demographic, clinical, and laboratory findings of the diagnosed agammaglobulinemia patients.

Pt’s no.	Sex	Age	Age of onset	History of infection	BTK protein expression	CD19 + B cell (%)	Serum IgM (gm/l)	Serum IgG (gm/l)	Serum IgA (gm/l)	Family history
P8	M	14y	1 m	Fever, RTI	390.0	**0.02** (11.9–21.0)	**<0.169** (0.45–2.44)	**3.45** (5.09–15.80)	**<0.256** (0.52–3.19)	−
P9	M	3y	7 m	Fever, RTI, Diarrhea	561.0	**0.1** (12.9–29.2)	**<0.169** (0.37–1.84)	**1.34** (2.95–11.56)	**<0.256** (0.27–2.46)	**+**
P10	M	6y	3y	Fever, RTI	424.0	**0.1** (17–37.2)	**<0.169** (0.37–2.24)	**2.41** (3.86–14.70)	**<0.256** (0.29–2.56)	−
P11	M	14y	5y	Fever, RTI, Diarrhea,	394.0	**0.5** (11.9–21.0)	**<0.169** (0.45–2.44)	**3.23** (5.09–15.80)	**<0.256** (0.52–3.19)	−
P12	M	8y	5y	Fever, RTI	435.00	**<0.02** (17–37.2)	**<0.169** (0.38–2.51)	**1.88** (4.62–16.82)	**<0.256** (0.34–2.74)	+
P13	M	18y	5y	Fever, RTI	593.00	**0.1** (11.9–21.0)	**<0.169** (0.49–2.01)	**1.34** (4.87–13.27)	**<0.256** (0.60–3.37)	−
P14	M	8y	6 m	Fever, RTI	492.0	**0.1** (17–37.2)	**<0.169** (0.38–2.51)	**3.48** (4.62–16.82)	**<0.256** (0.34–2.74)	−
P15	F	4y	6 m	RTI, Diarrhea	1203.0	**0.1** (12.9–29.2)	<**0.169** (0.37–1.84)	7.19 (2.95–11.56)	<**0.256** (0.27–2.46)	

+: history of maternal male family members who died due to infections in early childhood; y: year; m: month; F: female; M: male. The bold values indicate significant results.

Table 3 shows TACI/TNFRSF13B gene mutations in the diagnosed CVID patients. The analysis of exons 1 to 5 revealed no pathogenic variants in any of the patients. All seven patients expressed the same nucleotide substitution, resulting in benign variants in exons 2 and 5. In exon 2, there was a nucleotide substitution c.81G > A, which led to the benign variant p.Thr27=. The nucleotide substitution c.831 T > C in exon 5 resulted in the benign variation p.Ser277=. In both cases, the molecular consequence was synonymous. In P1 and P3, another synonymous variant was found in the 3 prime un-translated region (UTR) of exon 5, which occurred due to the nucleotide substitution c.*173G > A. In P3, a nucleotide substitution, c.752C > T, was found in exon 5, leading to a missense mutation, p.pro251leu, which was also classified as benign.

**Table 3:** TACI/TNFRSF13B gene variants in the diagnosed CVID patients.

Patient’s no.	Exon	Nucleotide substitutions	Amino acid change	Type of mutation	Classification	Geographic distribution
P1	2	c.81G > A	p.Thr27=	Synonymous	Benign	Germany, USA,
	5	c.831 T > C	p.Ser277=	Synonymous	Benign	No publications were found
		c.*173G > A		3 prime UTR	Benign	No publications were found
P2	2	c.81G > A	p.Thr27=	Synonymous	Benign	Germany, USA
	5	c.831 T > C	p.Ser277=	Synonymous	Benign	No publications were found
P3	2	c.81G > A	p.Thr27=	Synonymous	Benign	Germany, USA
	5	c.831 T > C	p.Ser277=	Synonymous	Benign	No publications were found
		c.752C > T	p.pro251leu	Missense	Benign	No publications were found
		c.*173G > A		3 prime UTR	Benign	No publications were found
P4	2	c.81G > A	p.Thr27=	Synonymous	Benign	Germany, USA
	5	c.831 T > C	p.Ser277=	Synonymous	Benign	No publications were found
P5	2	c.81G > A	p.Thr27=	Synonymous	Benign	Germany, USA
	5	c.831 T > C	p.Ser277=	Synonymous	Benign	No publications were found
P6	2	c.81G > A	p.Thr27=	Synonymous	Benign	Germany, USA
	5	c.831 T > C	p.Ser277=	Synonymous	Benign	No publications were found
P7	2	c.81G > A	p.Thr27=	Synonymous	Benign	Germany, USA
	5	c.831 T > C	p.Ser277=	Synonymous	Benign	No publications were found

Table 4 shows BTK gene mutations alongside flow cytometric findings in the diagnosed XLA patients. The analysis of exons 2 to 19 revealed seven pathogenic/likely pathogenic mutations in seven patients, including four nonsense (c.763C > T, c.1899C > T, c.1573C > T, and c.829G > T) and three missense mutations (c.863G > C, c.862C > T, and c.110 T > C).

**Table 4:** BTK gene mutations in the diagnosed XLA patients.

Pt’s no.	Exon	Domain	Nucleotide substitutions	Amino acid change	Type of mutation	Classification	Geographic distribution	Accession no.
P8	10	SH2	c.863G > C	p.Arg288Pro	Missense ^N^	Pathogenic	No submitter was found	PP107932
	18	SH1	c.1899C > T	p.Cys633=	Synonymous	Benign	Vietnam, USA, Austria, UK	PP547986
	19	SH1	c.*192G > A		3 prime UTR	Benign	No publications were found	
P9	8	SH3	c.763C > T	p.Arg255Ter	Nonsense	Pathogenic	USA, China, Japan,	PP555599
	19	SH1	c.*116A > C		3 prime UTR	Benign	No publications were found	
P10	2	PH	c.37C > T	p.Arg13Ter	Nonsense	Pathogenic	China, Spain, USA, Brazil, Japan, Greece	PP565372
	18	SH1	c.1899C > T	p.Cys633=	Synonymous	Benign	Vietnam, USA, Austria, UK	PP547987
	19	SH1	c.*192G > A		3 prime UTR	Benign	No publications were found	
P11	16	SH1	c.1573C > T	p.Arg525Ter	Nonsense	Likely pathogenic	Sweden	PP601381
	19	SH1	c.*116A > C		3 prime UTR	Benign	No publications were found	
P12	10	SH2	c.862C > T	p.Arg288Trp	Missense	Pathogenic/ Likely pathogenic	Mexico, USA, Australia, Italy, Taiwan	PP565373
	19	SH1	c.*116A > C		3 prime UTR	Benign	No publications were found	
P13	2	PH	c.110 T > C	p.Leu37Pro	Missense ^N^	Pathogenic	No submitter was found	PP107933
	19	SH1	c.*192G > A		3 prime UTR	Benign	No publications were found	
P14	9	SH3	c. 829G > T	p.Glu277Ter	Nonsense ^N^	Pathogenic	No submitter was found	PP601380
	18	SH1	c.1899C > T	p.Cys633=	Synonymous	Benign	Vietnam, USA, Austria, UK	PP547988

N: Novel mutation; PH: Pleckstrin homology; SH3: Src homology 3 domain; SH2: Src homology 2 domain; SH1: kinase domain.

## Discussion

**Figure 4:**
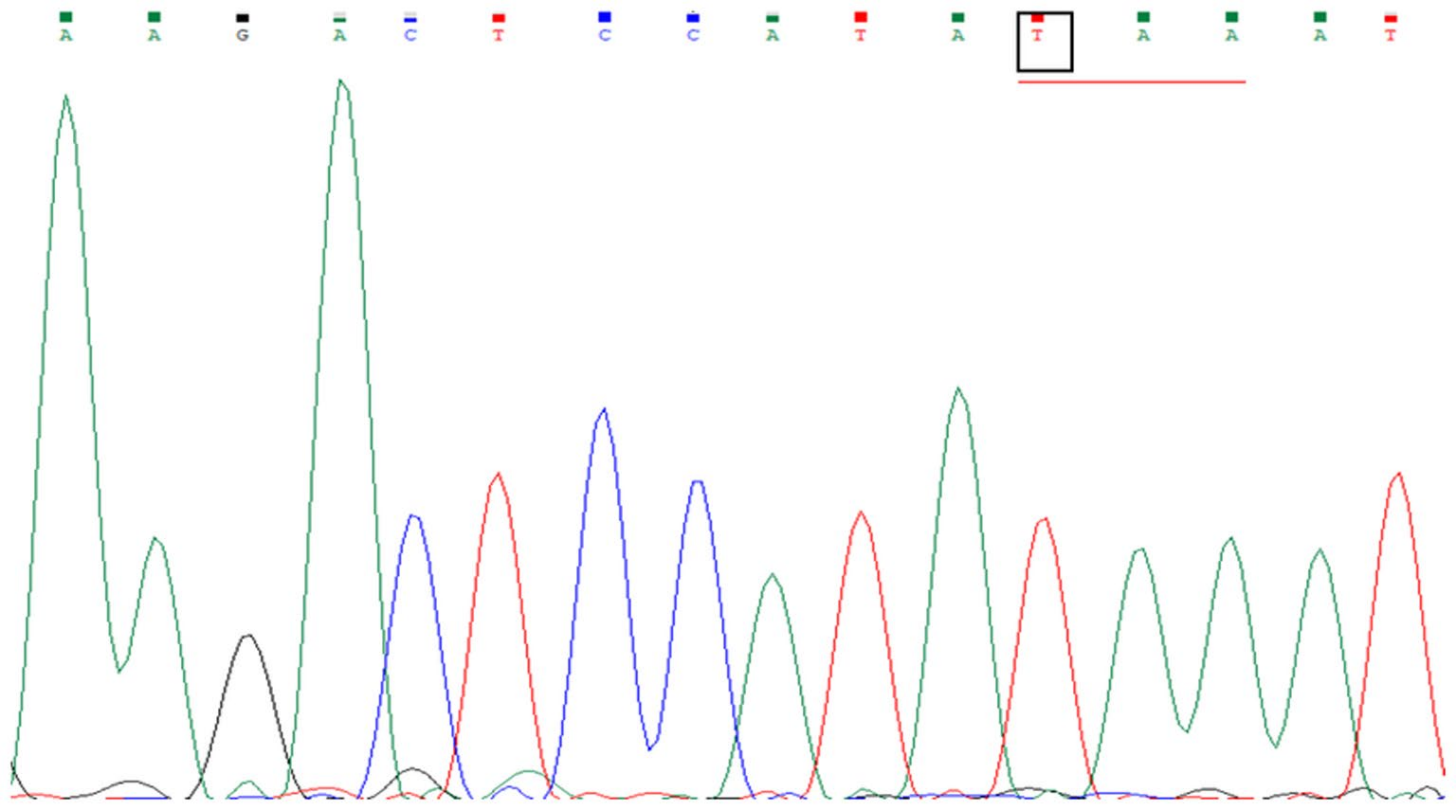
Sequence chromatogram of BTK variants in patient P14 in exon 9. A single-nucleotide substitution from G to T, c.829G *>* T, in exon 9 of the *BTK* gene was identified in the patient, resulting in a nonsense mutation. The codon GAA, encoding glutamate, was changed to the stop codon TAA.

**Figure 5:**
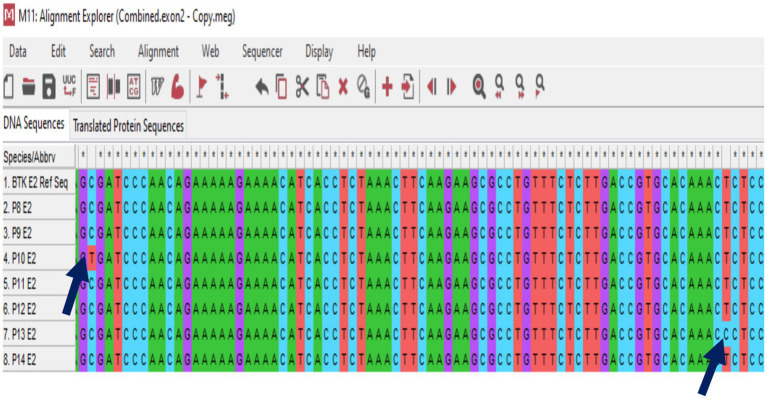
Multiple sequence analysis (MSA) of the FASTA sequence of exon 2 from seven XLA patients to detect mutations. Nucleotide substitutions are indicated by the blue arrows.

In this study, genetic analysis of the TACI/TNFRSF13B gene was performed on seven patients diagnosed with CVID. The TACI/TNFRSF13B gene is located at 17p11.2 and has 5 exons. Exon 1 to 5 analysis revealed no pathogenic variant in any CVID patients. In exon 2, the expressed benign variant p.Thr27 = has been previously described in several studies (10, 29, 30). There is no submitter for the synonymous variant p.Ser277 = in the NCBI GenBank, which was discovered in exon 5. Moreover, the synonymous variant, c.*173G > A, and the missense mutation, p.Pro251Leu, also have no submitter in the NCBI GenBank. The development and progression of CVID are believed to be influenced by environmental variables and epigenetic alterations, even in the absence of recognized pathogenic mutations. Patients with CVID might have a variety of clinical symptoms, even if they share comparable genetic origins. This suggests that other factors, including non-genetic ones, contribute to the disease’s heterogeneity, as 85–90% of cases of CVID have no underlying genetic cause. Therefore, the absence of a pathogenic mutation in CVID patients might seem to lessen the implications of the condition, but it does not diminish the significant clinical burden and the need for ongoing research into the disease’s complex etiology and pathogenesis (31). Moreover, only the TACI gene was analyzed in this study. Therefore, detection of other genes commonly associated with CVID (ICOS, CD19, BAFF-R) using genotypic and phenotypic methods could have increased the detection rate of CVID patients (8–14). Figure 4 shows the sequence chromatogram of BTK variants of P14 in exon 9, and Figure 5 shows multiple sequence analysis (MSA) of the FASTA sequence of exon 2 of seven XLA patients to detect mutation.

The BTK gene is located at Xq21.3-Xq22 and spans 37.5 kb, comprising 19 exons. This gene has a non-coding region as its first exon, while the next 18 exons encode the BTK protein (32). The Human Gene Mutation Database recorded 1,059 BTK gene mutations connected to XLA (33). Missense mutations are the most prevalent, alongside nonsense mutations, splice site mutations, insertions, and deletions. This study found seven pathogenic/likely pathogenic mutations and three benign variants in seven XLA patients. The pathogenic mutations included four nonsense (42.86%) and three missense mutations (57.14%). The results of the current study are in line with those of a previous study (34), which reported 58% recurrent mutations in a group of 30 patients.

The genetic profiles of seven XLA patients in this study also showed that the identified mutations spanned all domains of BTK, except for the TH domain. The PH, SH3, and SH2 domains each accounted for two mutations (28.57%) of all mutations, and one mutation (14.29%) was found in the SH1 domain. Missense mutations were found in the PH and SH2 domains, with a propensity for the SH2 domains. Nonsense mutations were found in the PH, SH3, and SH1 domains, with a propensity for SH3 domain. Therefore, it is noteworthy to mention that BTK mutations can occur sporadically (35–40).

A total of three-point mutations (c.863G > C, c.110 T > C, and c.829G > T) were found to be *de novo* in our patient. No submitter was found in the NCBI GenBank for this mutation. In the case of P8, a novel missense mutation, c.863G > C, was found, where a G-to-C transition at nucleotide position 863 in the BTK gene results in p.Arg288Pro (arginine is replaced with tryptophan). Arg288 is located within the BTK protein’s SH2-like domain. This mutation disrupts the interaction of the BTK protein with phosphotyrosine, leading to impaired B-cell function.

P13 showed a novel missense mutation, c.110 T > C, where a T-to-C transition at nucleotide position 110 in the BTK gene occurs in the PH domain of the BTK protein. The PH domain is known to be involved in protein–protein interactions and signaling pathways that are crucial for its overall structure and function. It results in the p.Leu37Pro mutation, where leucine is replaced with proline, leading to a block in B cell development.

In the case of P14, another novel nonsense mutation, c.829G > T, was detected, where a G-to-T transition occurs at nucleotide position 829 in the BTK gene. Here, p.Glu277Ter indicates that glutamic acid is replaced by a stop codon. Glu277 (glutamic acid at position 277) in the BTK gene is located within the SH3 domain, a region involved in protein–protein interactions. As nonsense mutation results in chain termination, producing truncated BTK proteins, and the clinical and laboratory findings align with XLA, this novel mutation can be considered pathogenic.

In the cases of P9, P10, P11, and P12, the identified point mutations were c.763C > T, c.1899C > T, c.1573C > T, and c.862C > T, respectively [accession no. CM940188, CM940182, CM950171, and CM940189 (41)]. c.763C > T, which is located in the SH3 domain, has been previously described as pathogenic in several studies (36, 37, 42–47). c.1899C > T, located in the PH domain, has also been mentioned previously (43, 48–54). VoRechovsky et al. (55) also found this c.1573C > T mutation among Swedish people, which was found in our P11. Another mutation, c.862C > T, found in P12 and located in the SH2 domain of the BTK protein, has previously been described in several studies (36, 42, 43, 56–61). All this information was collected from the NCBI GenBank (62–65).

In patients P8, P10, and P14, a synonymous variant, Cys633=, was found in the SH1 domain of the BTK protein. This variant has been previously described in several studies (42, 43, 56, 66, 67) cited in the NCBI GenBank (68). However, there is no functional evidence in ClinVar for this variation; therefore, it was considered benign.

In P8, P10, and P13, a benign variant, c.*192G > A, was found in the 3 prime UTR of the SH1 domain. Another benign variant, c.*116A > C, was also found in P9, P11, and P12, which lies in the 3 prime UTR of the SH1 domain. These variants were observed during a predisposition screen in an apparently healthy population. There are no published data in ClinVar for this variation. Allele frequency data from public databases do not support these variants as disease-causing. Therefore, these variants were classified as benign.

These findings suggest that genetic analysis of the BTK gene and evaluation of the immune function of suspected patients can increase the diagnosis rate. The results of this genetic analysis will help clinicians perform IVIG replacement therapy in a timely manner, potentially significantly reducing the incidence of complications and mortality rates. Genetic analysis of the BTK gene also has the potential to identify other patients and carriers within the patient’s family, thereby contributing to broader health outcomes and the practice of genetic counseling.

## Conclusion

This study identified CVID and XLA as the most prevalent predominantly antibody deficiencies. The TACI/TNFRSF13B gene analysis in CVID patients did not reveal any pathogenic variants. In the XLA patients, the mutations in the BTK gene were found to be diverse, with nonsense mutations being the most prevalent and showing a propensity for the SH3 domain. A total of three *de novo* point mutations (c.863G > C, c.110 T > C, and c.829G > T) were found in our patient. We identified that, in the majority of the cases, the mutation profile of our country is similar to that of the rest of the world. This diversity in mutations underscores the complexity of XLA in Bangladesh and the importance of mutation analysis in characterizing the BTK gene in XLA patients and of subsequent genetic counseling.

## Limitations

The limitations of the study are outlined as follows:

- Population-based studies involving a large number of samples in peripheral settings could not be performed due to limitations in time, budget, and resources.
- Additional flow cytometric markers, such as CXCR5, CD21, and CD38, which can increase the diagnostic rate of CVID, could not be included in this study due to budget constraints.
- Analysis of other genes in the signal transduction pathway of B cell development, such as *μ* heavy chain, Igα (CD79A), Igβ (CD79B), λ5 (IGGL1), and B-cell linker protein (BLNK), could not be performed due to limitations in time, budget, and resources.

## Data Availability

The datasets presented in this study can be found in online repositories. The names of the repository/repositories and accession number(s) can be found in the article/Supplementary material.
