# Supplementary material for: TACI and BTK gene analysis in predominantly antibody deficiencies among the primary immunodeficiency disorder patients in Bangladesh
Source: Front Med (Lausanne). 2025 Sep 25;12:1569810. doi: 10.3389/fmed.2025.1569810 (PMC12507743; doi:10.3389/fmed.2025.1569810)
Supplement: Supplementary file 1 [file Table_1.DOCX]

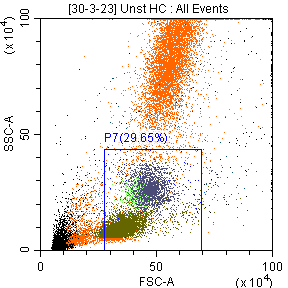

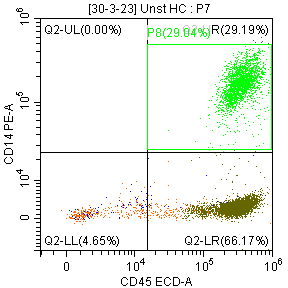

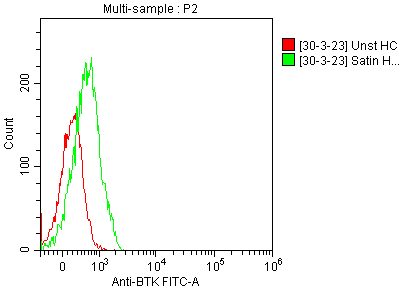


Stain

Unstain

| Name | Events | % Parent | Anti-BTK FITC-A MFI |
| --- | --- | --- | --- |
| Without Anti-BTK protein | 1019 | 5.98 | 578 |
| With Anti-BTK: Monocyte | 1177 | 4.81 | 1225 |

**In Healthy Control**


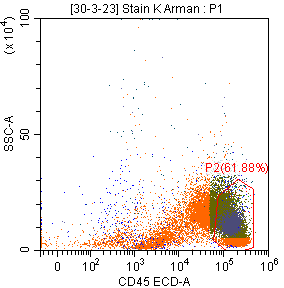

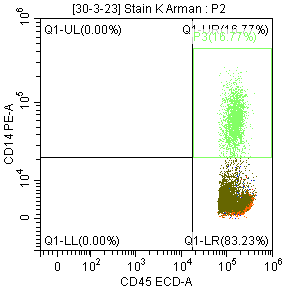

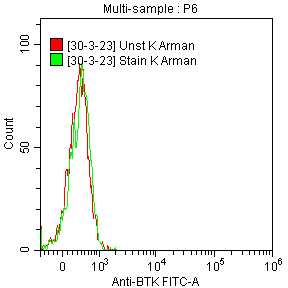


| Name | Events | % Parent | Anti-BTK FITC-A MFI |
| --- | --- | --- | --- |
| Without Anti-BTK protein | 507 | 18.06 | 309 |
| With Anti-BTK: Monocyte | 596 | 20.93 | 394 |

**In XLA Patient**

**Figure: Flow cytometric evaluation of intracellular BTK protein expression (MFI) in monocytes in a healthy control and a patient with X-linked agammaglobulinemia (XLA).**
